# *HIF-1α* rs11549465 C>T polymorphism contributes to increased cancer susceptibility: Evidence from 49 studies

**DOI:** 10.7150/jca.35716

**Published:** 2019-10-15

**Authors:** Hu-Nian Li, Ting He, Yong-Jiu Zha, Fang Du, Jie Liu, Hui-Ran Lin, Wen-Zi Yang

**Affiliations:** 1 Emergency and Critical Care Center, Renmin Hospital, Hubei University of Medicine, Shiyan 442000, Hubei, China.; 2 Department of Neurology, Renmin Hospital, Hubei University of Medicine, Shiyan 442000, Hubei, China.; 3 Animal Experimental Management Center, Public Technology Service Platform, Shenzhen Institutes of Advanced Technology, Chinese Academy of Sciences, Shenzhen 518055, Guangdong, China.

**Keywords:** *HIF-1α*, rs11549465 C>T, polymorphism, cancer, susceptibility

## Abstract

HIF-1*α* (hypoxia-inducible factor-1*α*) is a transcriptional factor that participates in the regulation of oxygen homeostasis. Despites numbers of case-control studies working on this area, the actual relationship of *HIF-1α* gene generic variant rs11549465 C>T imposing on cancer susceptibility remains unveiled. To get a better understanding of such relationship, this meta-analysis was carried out by incorporating all eligible case-control studies. Qualified articles were acquired from PubMed, CNKI, EMBASE, PMC, and Wanfang database update to April 2019. Odds ratios (ORs) and their corresponding 95% confidence intervals (CIs) were employed to estimate the relationship of interest. Heterogeneity tests, sensitivity analyses and publication bias assessments were also carried out to ensure the strength of our conclusion. A total of 46 articles with 49 studies including 12920 cases and 13363 controls were included. The results indicated that *HIF-1α* rs11549465 C>T was significantly related to the increased risk of overall cancer under four genetic models (TT vs. CC: OR=2.06, 95% CI=1.34-3.16; TT vs. CC/CT: OR=2.42, 95% CI=1.60-3.65; CT/TT vs. CC: OR=1.21, 95% CI=1.04-1.40; T vs. C: OR=1.29, 95% CI=1.12-1.48). Furthermore, enhanced cancer risk was detected after stratification by cancer type, ethnicity, the source of controls and HWE. These results suggest that *HIF-1α* rs11549465 C>T polymorphism may predispose to cancer susceptibility.

## Introduction

Cancer ranks itself the leading causes of death around the world. In 2019, 1,762,450 new cancer cases and 606,880 cancer deaths are projected to occur in the United States. It has become a universal public health issue 1. The most distinguished feature of cancer, un-controlled cell proliferation being one of them, is that it can assault the other vicinal parts of the body and diffuse to other organs. We refer this process to metastases, and this process could later evolve into a major cause of death from cancer. The exact etiology of carcinogenesis has not been fully verified 2. More and more evidence point to genetic variation in contributing to the initiation and progression of cancer 3, 4. However, due to cancer's complexity in nature, with heterogeneity being one of is feature, identification of this susceptibility is still a puzzle for us and most correlation has not been ascertained. On the other hand, during the decades, it has become universally agreed that single nucleotide polymorphisms (SNPs) are a common type of genetic variations that is the most frequently studied in connection with cancer susceptibility and that it consequently can act as the markers of many cancers 5.

Hypoxia possesses a vital role in the maintenance of tumor microenvironments. Hypoxic tumor microenvironment triggers extensive cellular responses, such as angiogenesis, proliferation and invasion 6. By adjusting the oxygen pressure that results in gene alteration, hypoxia may control tumor cell phenotypes 6. Hypoxia-inducible factor 1 (HIF-1) is a major transcriptional regulator implicated in homeostasis of oxygen. Koshiji et al. illustrated that HIF-1 leads to genetic instability by restraining the DNA mismatching repair system (MSH2 and MSH6) 7. HIF-1 is a dimeric protein complex that possesses two components known as α and β subunits 8. Studies have demonstrated that HIF-1*α* plays a vital role in activating various genes that is significantly involved with cell adhesion, erythropoiesis, angiogenesis and glucose transportation in the process of cancer development and progress 9.

Mounting evidence provided that featuring a high tumor grade, HIF-1α is over-stated in numbers of human cancers, indicating that HIF-1α functions as an independent element of cancer prognosis 10. *HIF-1α* has been a research hot spot and numerous SNPs in *HIF-1α* were identified, whose polymorphism known as 1772 C>T (rs11549465 C>T, Pro582Ser), having been the most widely investigation polymorphism. rs11549465 C>T is a nonsynonymous SNP. Compared to the wild type, this polymorphic variant can tremendously enhance transcriptional activity in both normoxic and hypoxic environment in *in-vitro* studies 11. Moreover, *HIF-1α* rs11549465 C>T is linked to increased tumor microvessel density which makes contribution to the cancer progression. *HIF-1α* rs11549465 C>T polymorphism was previously investigated in various types of cancer. Nevertheless, the conclusions obtained from previous epidemiological studies are inconsistent and contradictory. Thus, the relationship between *HIF-1α* rs11549465 C>T polymorphism and cancer risk requires further exploration. Herein, we performed this more comprehensive meta-analysis on selected case-control studies in the aim of giving a more thorough demonstration of the association of *HIF-1α* rs11549465 C>T polymorphism with cancer risk.

## Materials and Methods

### Publication search

We systematically searched EMBASE, PubMed, PMC, Wanfang and CNKI to retrieve relatively pertinent publications based on case-control studies (update to March 18, 2019). No language restriction is made for this analysis. The search terminology involved were as listed: 1) *hypoxia-inducible factor-1* or* HIF-1α* or rs11549465 or 1772 C>T; 2) SNPs or polymorphisms or polymorphism or variants; 3) cancer or carcinoma or neoplasm or tumor. To acquire all qualified publications, we also reviewed the references of the selected studies.

### Eligibility criteria

Impertinent and irrelevant studies were excluded on primary stage. Elimination criteria were: if 1) the study population was not mapped out; 2) it is not case-control study; 3) lack of information in allele frequency. Other than that, editorials, reviews and meta-analysis were ruled out. Only case-control studies with detailed number of different genotypes for estimating odds ratios (ORs) with 95% confidence intervals (CIs) were taken into the final analysis.

### Data extraction

Two authors (Hu-Nian Li and Ting He) were arranged to extract information of all the articles respectively. Items listed below were extracted from every single study: 1) authors name; 2) publication year; 3) ethnicity of the study subject; 4) cancer type; 5) allelic frequency; 6) quality score. Studies with scores ≤9 were of low quality, whereas those with scores >9 were of high quality 12, 13. All the disputable parts were compromised by discussion before consensus was made finally.

### Statistical methods

We first performed Hardy-Weinberg equilibrium (HWE) for the controls utilizing the goodness-of-fit test. Homozygous model, heterozygous model, recessive model, dominant model, and allele model were employed to determine the relationship between *HIF-1α* rs11549465 C>T polymorphism and cancer risk by calculating ORs with the corresponding 95% CIs. Moreover, we conducted the stratification analysis by ethnicity, cancer type, source of control, and HWE in controls. We also used Chi square-base *Q*-test to gauge the presence of heterogeneity. The fixed-effect model was used to compute the pooled OR, given the studies were confirmed to be homogeneous (*P*>0.10 for the *Q* test). Or the random-effect model should be used instead. Sensitivity analysis was undertaken on the base of re-calculation of the ORs and 95% CIs by excluding each study individually. In order to detect the presence of publication bias, Begg's funnel plot and Egger's linear regression were adopted simultaneously. We also performed the trial sequential analysis (TSA) to avoid the random errors caused by repeated significance testing and dispersed data 13. Version 11.0 STATA (Stata Corporation, College Station, TX) was selected to generate all statistical analysis. All the statistics were two-sided with *P* value <0.05 as a baseline significant finding.

## Results

### Study characteristics

**Figure 1:**
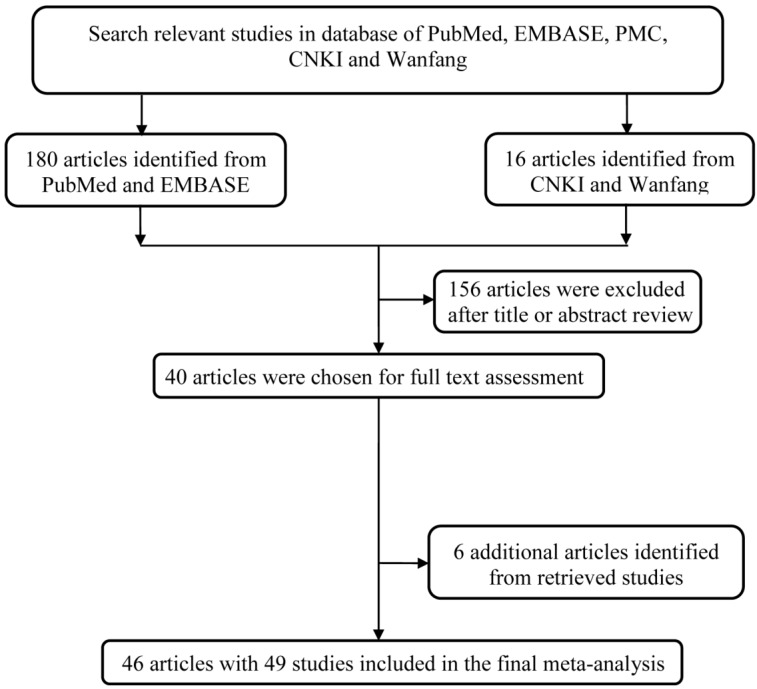
The main flowchart of this work.

The study workflow was graphically displayed in **Figure 1**. We first collected 196 articles of the interest by a comprehensive search in the above-mentioned databases. After a basic check-up on articles relevance and abstracts conciseness, 156 articles were ruled out, which left us a total of 40 articles for full text assessment. To expand its sample size to ensure statistical representativeness, we identified another 6 articles from retrieve studies, quantity adding up to 46 articles in total 14-59. Ultimately, 46 articles with 49 studies were contained in this analysis. A total of 12920 cases and 13363 controls was enrolled into this study for analyzing (**Table 1)**.

### Quantitative analysis

**Figure 2:**
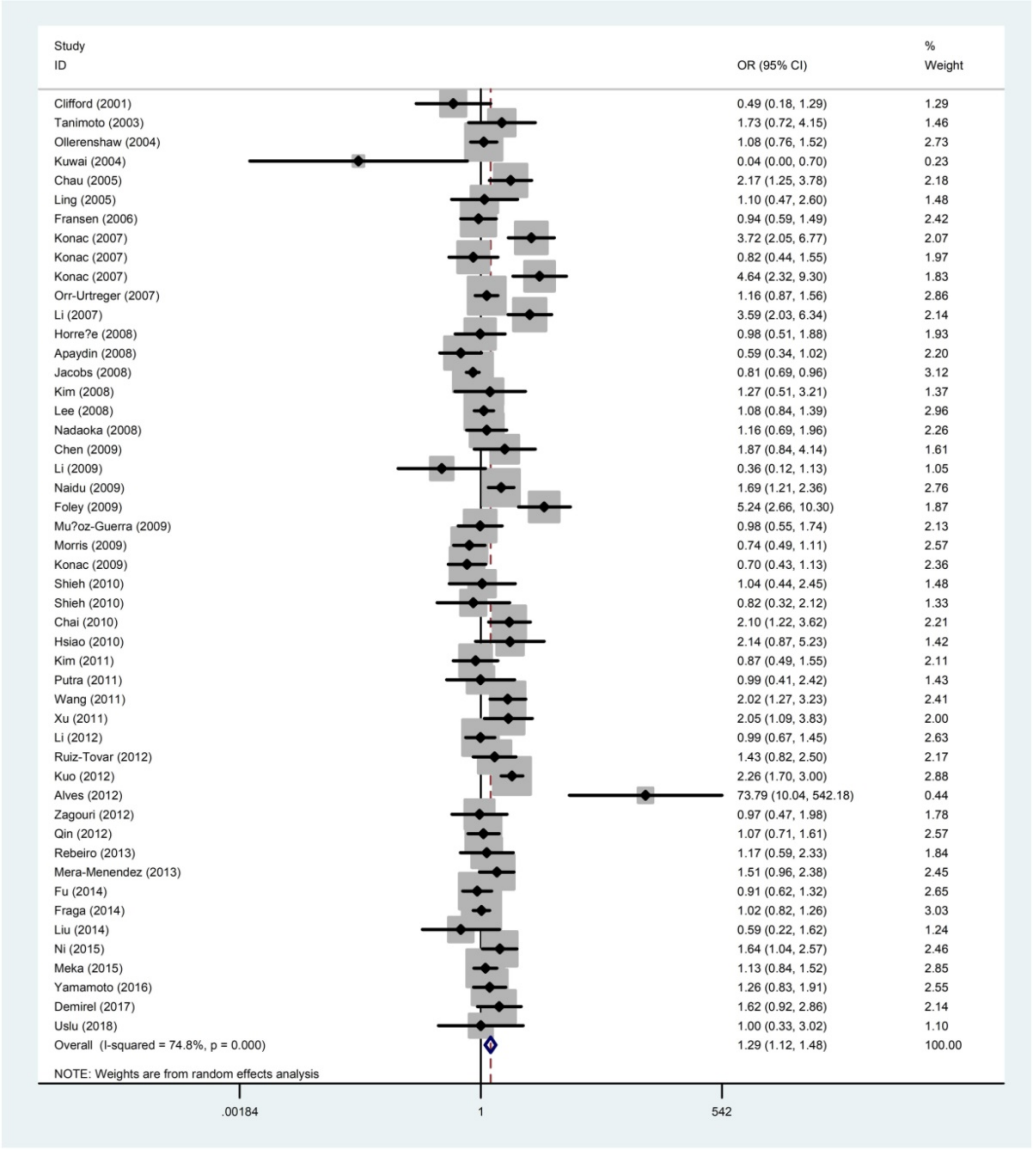
Forest plot for the correlation between the *HIF-1α* rs11549465 C>T polymorphism and cancer susceptibility under the allele comparison model. The horizontal lines represent the study-specific ORs and 95% CIs. The diamond represents the pooled results of OR and 95% CI.

The quantitative results of the meta-analysis were displayed in **Table 2** and **Figure 2**. The results concluded that the rs11549465 C>T polymorphism was significantly related to the increased risk of overall cancer under homozygous model (TT vs. CC: OR=2.06, 95% CI=1.34-3.16), recessive model (TT vs. CC/CT: OR=2.42, 95% CI=1.60-3.65); dominant model (CT/TT vs. CC: OR=1.21, 95% CI=1.04-1.40), and allele model (T vs. C: OR=1.29,95% CI =1.12-1.48). We failed to detect any distinguished relationship between rs11549465 C>T and renal cell carcinoma (RCC), endometrial cancer, colorectal cancer, lung cancer, breast cancer, hepatocellular cancer (HCC) under all the five genetic models. However, we observed that the rs11549465 C>T polymorphism could confer to increased risk in subgroups of prostate cancer (CT vs. CC/CT: OR=1.51, 95% CI=1.01-2.26; CT/TT vs. CC: OR=1.56, 95% CI=1.04-2.34; T vs. C: OR=1.54, 95% CI =1.05-2.25), cervical cancer (TT vs. CC: OR=7.63, 95% CI=1.83-31.8; TT vs. CC/CT: OR=6.60, 95% CI=2.07-21.0), oral cancer (TT vs. CC: OR=2.61, 95% CI=1.19-5.72; TT vs. CC/CT: OR=13.2, 95% CI=1.08-162), pancreatic cancer (TT vs. CC: OR=3.39, 95% CI=1.28-8.97; TT vs. CC/CT: OR=2.42, 95% CI=1.60-3.65) and other cancers (TT vs. CC: OR=2.62, 95% CI=1.24-5.55; TT vs. CC/CT: OR=2.64, 95% CI=1.26-5.56; T vs. C: OR=1.28, 95% CI=1.00-1.62).

When it comes to the stratification analysis by the ethnicity, significant increased risk was detected in Asians, Caucasians and mixed population. In terms of source of controls, either population-based controls or hospital-based controls were associated with the increase risk of cancer. Further subgroup analysis by HWE in controls revealed that no significant correlation was observed in subgroup of HWE≤0.05. As regard to the quality of publications, significant increased risk was detected in high-quality and low-quality publications.

### Heterogeneity and sensitivity analysis

**Figure 3:**
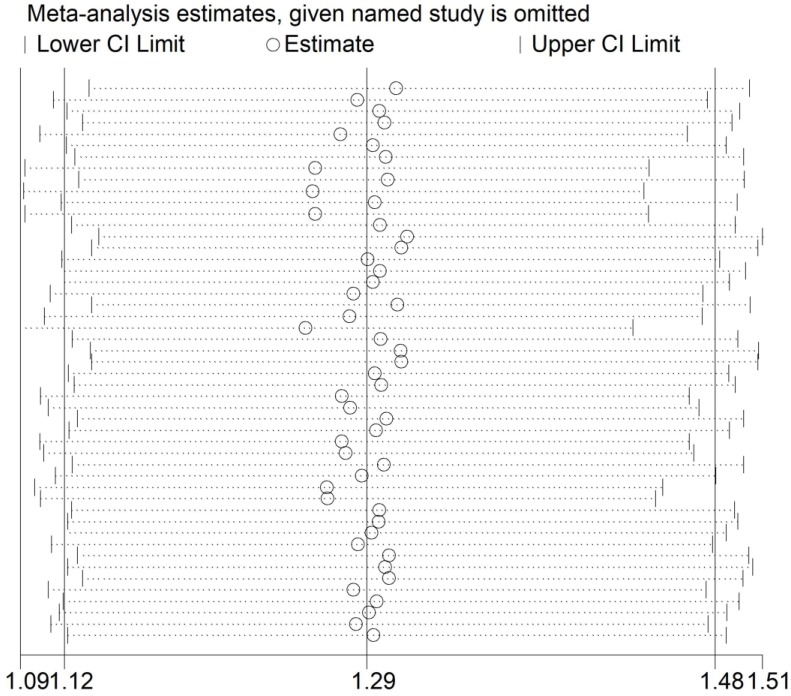
Sensitivity analysis of the association between *HIF-1α* rs11549465 C>T and cancer susceptibility. Each point represents the recalculated OR after deleting a separate study.

The *Q* test (*P*<0.001) implied an existence of heterogeneity under all the genetic models. Thus, we adopted a random-effect model to produce ORs and 95% CIs. In addition, the sequential sensitivity analysis was performed to give an evaluation of the impact of a sole study on the pooled estimation. Given the attempt of omitting in each study incurred no statistical fluctuation of the pooled ORs, we have reason to believe that the meta-analysis's data is of great reliability (**Figure 3**).

### Publication bias

**Figure 4:**
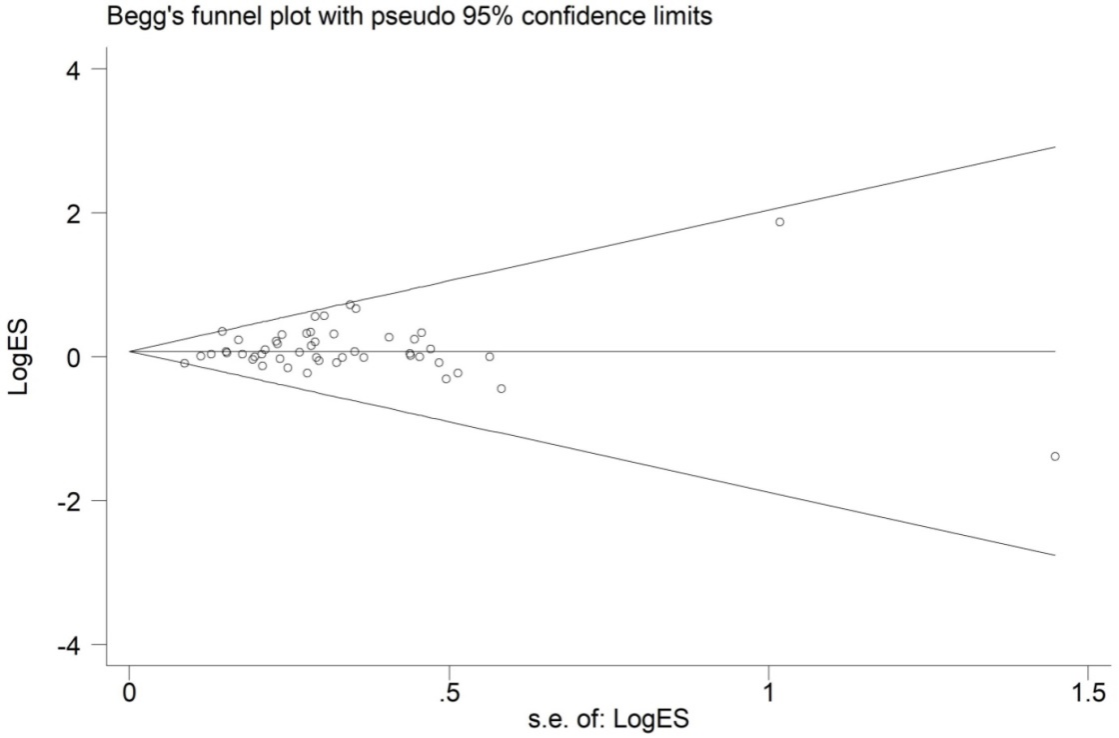
Funnel plot analysis to detect publication bias for *HIF-1α* rs11549465 C>T polymorphism under the allele comparison model. Each point represents a separate study.

From the shape of the Begg's funnel plot shown in **Figure 4**, no evidence of asymmetry was found. Egger's test's statistics also gives no evidence of publication bias among the studies.

### Trial sequential analysis (TSA)

**Figure 5:**
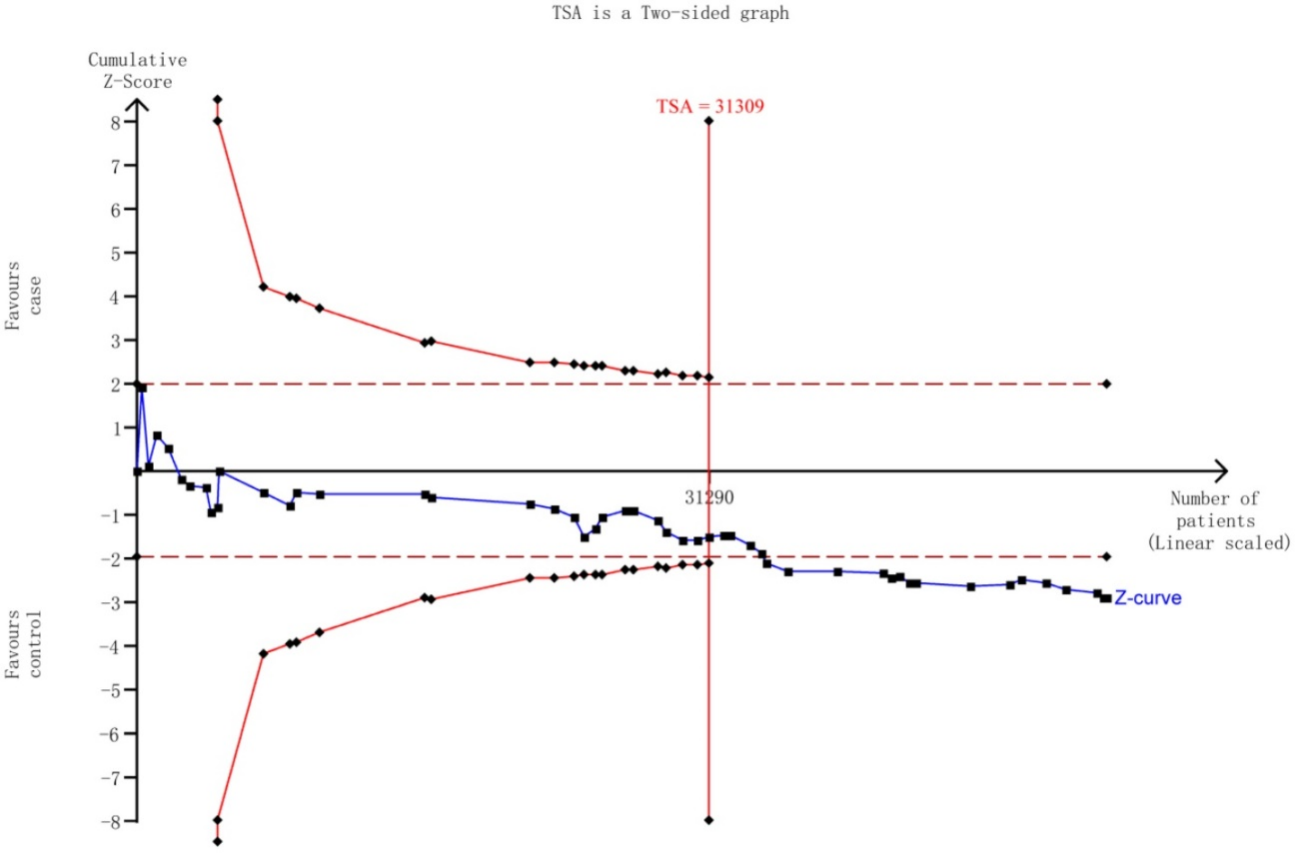
The required information size to demonstrate the correlation between *HIF-1α* rs11549465 C>T polymorphism with cancer susceptibility. The solid blue line is the cumulative Z-curve. The dashed inward-sloping line to the left represents the trial sequential monitoring boundaries.

The TSA showed that the cumulative z-curve did not cross both the traditional threshold and the TSA threshold, yet the accumulated information was sufficient, indicating that no further evidence was needed to verify the conclusion (**Figure 5**).

## Discussion

In the current meta-analysis, we systematically evaluate the relationship between *HIF-1α* rs11549465 C>T polymorphism and cancer risk by using 49 case-control studies. Our analysis showed that *HIF-1α* rs11549465 C>T polymorphism could increase risk of overall cancer risk and specific cancer risk. Among all the epidemical studies on the rs11549465 C>T polymorphism and cancer risk, this could be by now the most comprehensive one.

The *HIF-1α* gene is located at chromosome 14q21-24. *HIF-1α* regulates the expression of hundreds of genes which moderates the vital cellular functions like proliferation, apoptosis, angiogenesis, glucose metabolism, erythropoiesis, and iron metabolism 60. Due to the complex functional mechanism and regulatory roles of *HIF-1a* in hypoxic stress, the possible role of *HIF-1a* gene SNPs in cancer susceptibility has evoked intensive investigation. The most broadly studied *HIF-1α* polymorphism rs11549465 C>T (Pro582Ser) could induce proline-to-serine amino acid substitutions. However, the exact role of rs11549465 C>T polymorphism in cancer risk obtained from different studies remain inconclusive.

In 2001, Clifford et al. 14 carried out a first case-control study investigating the relationship between *HIF-1α* rs11549465 C>T and cancer risk. However, association analysis between rs11549465 C>T and RCC risk in panels of 20 cases and 44 non-neoplastic controls did not reveal allelic frequency differences. An investigation conducted by Konac et al. 21 using endometrial, ovarian, and cervical cancers in the Turkish population revealed that the rs11549465 C>T polymorphism of the *HIF-1α* may contribute to risk of endometrial and cervical cancers. In a meta-analysis performed by Zhao et al. 10 in 2009 using 5387 controls and 4131 cancer cases, the *HIF-1α* rs11549465 C>T polymorphism was reported to be related to increased cancer risk. In 2015, Li et al. 61 conducted an updated meta-analysis by enrolling 7807 cases and 8633 controls. They obtained a similar result that the *HIF-1α* rs11549465 C>T polymorphism predispose to higher overall cancer risk. To better illustrate the relationship of interest, we hereby conducted this updated meta-analysis by using all the qualified publications with a total of 12920 cases and 13363 controls. The results revealed that *HIF-1α* rs11549465 C>T polymorphism contributes to increased overall cancer risk. In a sense, this meta-analysis has succeeded in giving a clearer clue of the relationship between *HIF-1α* rs11549465 C>T polymorphism and cancer risk.

In the current meta-analysis, we undertaken many measurements to increase the credibility of our conclusion. First and foremost, we included as many as qualified studies to expand the analyzed sample size, by incorporating studies not only pressed in English but also in Chinese. Second, we adopted the sensitivity analysis and the publication bias. However, several limitations could not be settled down. First, between-study heterogeneity exists, which might weaken the persuasiveness of the conclusion. Second, the relationship strength was only assessed by use of unadjusted estimates. Lacking original data, such as environment factor, adjustment analysis was absent. Third, most of the included studies were conducted among Asians and Caucasians. The lack of other ethnicities, such as Africans, compromised the generalization of the conclusion.

In a word, our finding has come to a fruition that *HIF-1α* rs11549465 C>T polymorphism was significantly related to an increase in cancer risk. Our work no doubt will encourage more dedication into further elucidation of the etiology of cancer predisposition. However, with limited sample size of subgroup analysis, we must admit that this analysis is imperfect and thus in the future more case-control studies should be conducted with a larger size of samples.

## Figures and Tables

**Table 1:** Main characteristics of included studies in the meta-analysis

Surname	Year	Cancer type	Country	Ethnicity	Control source	Genotype method	Case				Control				HWE	Score
							CC	CT	TT	All	CC	CT	TT	All		
Clifford	2001	RCC	UK	Caucasian	PB	PCR	30	5	0	35	110	27	6	143	0.018	6
Tanimoto	2003	HNSCC	Japan	Asian	PB	PCR-Sequencing	45	10	0	55	98	12	0	110	0.545	5
Ollerenshaw	2004	RCC	UK	Caucasian	PB	PCR	16	54	90	160	1	90	71	162	<0.001	6
Kuwai	2004	Colorectal cancer	Japan	Asian	PB	PCR-Sequencing	100	0	0	100	89	11	0	100	0.561	7
Chau	2005	Prostate cancer	USA	Mixed	PB	PCR	161	29	6	196	179	14	3	196	0.002	6
Ling	2005	ESCC	China	Asian	PB	PCR-RFLP	84	11	0	95	93	11	0	104	0.569	6
Fransen	2006	Colorectal cancer	Sweden	Caucasian	PB	PCR-RFLP	167	28	3	198	213	43	2	258	0.916	8
Konac	2007	Cervical cancer	Turkey	Caucasian	HB	PCR-RFLP	10	14	8	32	68	37	2	107	0.229	7
Konac	2007	Ovarian cancer	Turkey	Caucasian	HB	PCR-RFLP	34	14	1	49	68	37	2	107	0.229	5
Konac	2007	Endometrial cancer	Turkey	Caucasian	HB	PCR-RFLP	4	12	5	21	68	37	2	107	0.229	5
Orr-Urtreger	2007	Prostate cancer	Israel	Caucasian	PB	PCR-RFLP	287	99	16	402	217	80	3	300	0.137	10
Li	2007	Prostate cancer	USA	Mixed	PB	PCR-RFLP	818	209	14	1041	175	13	0	188	0.623	10
Horre´e	2008	Endometrial cancer	Netherlands	Caucasian	PB	PCR	50	5	3	58	463	84	12	559	0.001	10
Apaydin	2008	Breast cancer	Turkey	Caucasian	PB	PCR-RFLP	79	21	2	102	68	29	5	102	0.415	6
Jacobs	2008	Prostate cancer	USA	Mixed	HB	MassARRAY	1156	252	12	1420	1138	284	28	1450	0.040	11
Kim	2008	Breast cancer	Korea	Asian	HB	PCR-Sequencing	81	8	1	90	93	9	0	102	0.641	9
Lee	2008	Breast cancer	Korea	Asian	PB	SNP-ITTM	1207	119	6	1332	1245	123	1	1369	0.250	11
Nadaoka	2008	Bladder cancer	Japan	Asian	PB	PCR-RFLP	197	21	1	219	419	42	0	461	0.350	10
Chen	2009	Oral cancer	China	Asian	PB	PCR-RFLP	163	10	1	174	334	13	0	347	0.722	9
Li	2009	Gastric cancer	China	Asian	PB	PCR-LDR	83	4	0	87	93	13	0	106	0.501	6
Naidu	2009	Breast cancer	Malaysia	Asian	PB	PCR-RFLP	294	100	16	410	222	50	3	275	0.922	10
Foley	2009	Prostate cancer	Ireland	Caucasian	PB	PCR-Sequencing	65	30	0	95	175	13	0	188	0.623	9
Muñoz-Guerra	2009	Oral cancer	Spain	Caucasian	PB	PCR	57	6	7	70	113	27	8	148	0.001	7
Morris	2009	RCC	UK	Caucasian	PB	Taqman	290	39	3	332	262	46	5	313	0.080	10
Konac	2009	Lung cancer	Turkey	Caucasian	HB	PCR-RFLP	110	31	0	141	111	43	2	156	0.335	8
Shieh	2010	OSCC	China	Asian	HB	PCR-Sequencing	282	23	0	305	89	7	0	96	0.710	8
Shieh	2010	Oral cancer	China	Asian	HB	PCR	187	12	0	199	89	7	0	96	0.710	8
Chai	2010	Cervical cancer	China	Asian	HB	PCR	65	25	7	97	94	21	2	117	0.520	8
Hsiao	2010	HCC	China	Asian	HB	PCR-RFLP	94	8	0	102	334	13	0	347	0.722	9
Kim	2011	Cervical cancer	Korea	Asian	HB	SNaPShot	177	22	0	199	187	27	0	214	0.325	9
Putra	2011	Lung cancer	Japan	Asian	HB	PCR-Sequencing	74	9	0	83	98	12	0	110	0.545	9
Wang	2011	Pancreatic cancer	China	Asian	HB	PCR-Sequencing	209	54	0	263	242	29	0	271	0.352	10
Xu	2011	Glioma cancer	China	Asian	HB	PCR-RFLP	121	27	2	150	135	14	1	150	0.354	8
Li	2012	Prostate cancer	China	Asian	HB	Taqman	612	48	2	662	659	57	0	716	0.267	10
Ruiz-Tovar	2012	Pancreatic cancer	Spain	Caucasian	PB	PCR	47	1	11	59	116	28	8	152	0.0016	9
Kuo	2012	Lung cancer	China	Asian	HB	PCR-RFLP	153	94	38	285	216	73	11	300	0.132	10
Alves	2012	Oral cancer	Brazil	Mixed	PB	PCR	0	1	39	40	0	85	3	88	<0.001	9
Zagouri	2012	Breast cancer	Greece	Caucasian	HB	PCR-RFLP	98	15	0	113	107	17	0	124	0.413	5
Qin	2012	RCC	China	Asian	HB	Taqman	572	46	2	620	578	43	2	623	0.220	10
Rebeiro	2013	Breast cancer	Portugal	Caucasian	PB	PCR-RFLP	74	21	1	96	61	7	4	72	0.001	8
Mera-Menendez	2013	Glottic cancer	Spain	Caucasian	HB	PCR	85	18	15	118	114	27	8	149	0.001	10
Fu	2014	Cervical cancer	China	Asian	HB	PCR	467	49	2	518	492	60	1	553	0.550	11
Fraga	2014	Prostate cancer	Portugal	Caucasian	HB	Taqman	566	156	14	736	579	164	11	754	0.400	11
Liu	2014	HCC	China	Asian	HB	PCR-RFLP	152	4	1	157	162	11	0	173	0.6658	9
Ni	2015	Digestive tract cancers	China	Asian	HB	PCR-RFLP	219	44	4	267	241	34	0	275	0.2745	10
Meka	2015	Breast cancer	India	Asian	HB	PCR	245	94	9	348	229	89	2	320	0.0322	10
Yamamoto	2016	Lung cancer	Japan	Asian	HB	TaqMan	405	55	2	462	341	37	1	379	0.9972	10
Demirel	2017	Colorectal cancer	Turkey	Caucasian	HB	ARMS-PCR	62	27	3	92	81	16	4	101	0.0144	8
Uslu	2018	Laryngeal Cancer	Turkey	Caucasian	HB	PCR	28	7	0	35	28	7	0	35	0.5109	5

HWE, Hardy-Weinberg equilibrium; PB, population based; HB, hospital based; RCC, renal cell carcinoma; HNSCC, head and neck squamous cell carcinoma; ESCC, esophageal squamous cell carcinoma; OSCC, oral squamous cell carcinoma; HCC, hepatocellular cancer; PCR-RFLP, polymerase chain reaction-restriction fragment length polymorphism.

**Table 2:** Meta-analysis of *HIF-1α* rs11549465 C>T polymorphism and cancer risk

Variables	Homozygous			Heterozygous			Recessive			Dominant			Allele	
	TT vs. CC			CT vs. CC			TT vs. CC/CT			CT/TT vs. CC			T vs. C	
	OR (95% CI)	*P* ^het^		OR (95% CI)	*P* ^het^		OR (95% CI)	*P* ^het^		OR (95% CI)	*P* ^het^		OR (95% CI)	*P* ^het^
All	**2.06 (1.34-3.16)**	<0.001		1.14 (0.99-1.33)	<0.001		**2.42 (1.60-3.65)**	<0.001		**1.21 (1.04-1.40)**	<0.001		**1.29 (1.12-1.48)**	<0.001
Cancer type														
RCC	0.37 (0.12-1.12)	0.282		0.64 (0.32-1.29)	0.012		1.31 (0.77-2.24)	0.350		0.66 (0.35-1.23)	0.024		0.92 (0.70-1.19)	0.252
Colorectal	1.30 (0.40-4.17)	0.579		0.83 (0.24-2.83)	0.005		1.18 (0.37-3.78)	0.465		0.86 (0.29-2.60)	0.008		0.92 (0.37-2.26)	0.019
Prostate	1.67 (0.66-4.19)	0.008		**1.51 (1.01-2.26)**	<0.001		1.62 (0.66-3.99)	0.011		**1.56 (1.04-2.34)**	<0.001		**1.54 (1.05-2.25)**	<0.001
Cervical	**7.63 (1.83-31.8)**	0.170		1.22 (0.76-1.96)	0.064		**6.60 (2.07-21.0)**	0.289		1.46 (0.78-2.72)	0.004		1.55 (0.80-3.02)	<0.001
Endometrial	9.06 (0.53-156.2)	0.014		1.69 (0.18-16.2)	0.003		5.85 (0.93-36.9)	0.086		2.29 (0.25-21.1)	0.001		2.12 (0.46-9.78)	0.002
Breast	1.38 (0.33-5.74)	0.045		0.99 (0.80-1.23)	0.329		1.38 (0.33-5.75)	0.044		1.02 (0.85-1.22)	0.458		1.04 (0.88-1.23)	0.434
Oral	**2.61 (1.19-5.72)**	0.514		1.06 (0.61-1.85)	0.081		**13.2 (1.08-162)**	<0.001		1.24 (0.79-1.93)	0.149		1.90 (0.88-4.07)	<0.001
Lung	1.92 (0.35-10.5)	0.103		1.19 (0.78-1.82)	0.044		1.93 (0.43-8.66)	0.154		1.23 (0.71-2.13)	0.002		1.23 (0.69-2.20)	<0.001
HCC	3.20 (0.13-79.1)	-		0.96 (0.17-5.29)	0.021		3.33 (0.14-82.2)	-		1.06 (0.24-4.68)	0.035		1.15 (0.33-4.06)	0.061
Pancreatic	**3.39 (1.28-8.97)**	-		0.50 (0.02-14.0)	0.001		**2.42 (1.60-3.65)**	-		1.39 (0.54-3.56)	0.032		**1.75 (1.23-2.51)**	0.349
Others	**2.62 (1.24-5.55**)	0.784		1.13 (0.87-1.47)	0.275		**2.64 (1.26-5.56)**	0.810		1.22 (0.95-1.57)	0.274		**1.28 (1.00-1.62)**	0.239
Ethnicity														
Caucasian	1.54 (0.81-2.87)	<0.001		1.01 (0.75-1.35)	<0.001		**1.82 (1.15-2.89)**	0.004		1.10 (0.84-1.44)	<0.001		1.21 (0.97-1.51)	<0.001
Asian	**4.07 (2.61-6.34)**	0.995		**1.19 (1.02-1.38)**	0.010		**3.67 (2.37-5.72)**	0.997		**1.25 (1.06-1.47**)	0.001		**1.28 (1.09-1.51)**	<0.001
Mixed	1.27 (0.26-6.15)	0.028		1.85 (0.70-4.86)	<0.001		7.57 (0.31-184)	<0.001		1.86 (0.67-5.16)	<0.001		**3.24 (1.02-10.3)**	<0.001
Source of control														
PB	1.61 (0.90-2.89)	0.014		1.03 (0.76-1.40)	<0.001		**2.51 (1.33-4.74)**	<0.001		1.12 (0.85-1.47)	<0.001		1.27 (0.99-1.62)	<0.001
HB	**2.61 (1.39-4.91)**	<0.001		**1.17 (1.00-1.36)**	0.001		**2.36 (1.33-4.18)**	<0.001		**1.25 (1.05-1.48)**	<0.001		**1.30 (1.09-1.55)**	<0.001
HWE														
>0.05	**2.92 (1.34-3.16)**	0.015		**1.20 (1.02-1.41)**	<0.001		**2.71 (1.76-4.16)**	0.111		**1.26 (1.06-1.50)**	<0.001		**1.30 (1.10-1.54)**	<0.001
≤0.05	1.18 (0.59-2.36)	<0.001		0.91 (0.62-1.33)	<0.001		2.10 (0.99-4.44)	<0.001		1.04 (0.78-1.38)	0.002		1.24 (0.95-1.63)	<0.001
Score														
>9	**2.26 (1.32-3.85)**	0.001		1.13 (0.97-1.32)	<0.001		**2.19 (1.32-3.63)**	0.004		**1.21 (1.02-1.43)**	<0.001		**1.25 (1.05-1.49)**	<0.001
≤9	1.76 (0.84-3.67)	<0.001		1.10 (0.83-1.47)	<0.001		**2.59 (1.31-5.14)**	<0.001		1.18 (0.90-1.54)	<0.001		**1.31 (1.03-1.67)**	<0.001

Het, heterogeneity; RCC, renal cell carcinoma; HB, hospital based; PB, population based.

## Abbreviations

HIF-1: hypoxia-inducible factor 1
ORs: odds ratios
Cis: confidence intervals
HWE: Hardy-Weinberg equilibrium
TSA: trial sequential analysis
